# Pulsed Arterial Spin Labeling and Segmented Brain Volumetry in the Diagnostic Evaluation of Frontotemporal Dementia, Alzheimer’s Disease and Mild Cognitive Impairment

**DOI:** 10.3390/tomography8010018

**Published:** 2022-01-17

**Authors:** Dominique Cornelius Marterstock, Michael Franz Xaver Knott, Philip Hoelter, Stefan Lang, Timo Oberstein, Johannes Kornhuber, Arnd Doerfler, Manuel A. Schmidt

**Affiliations:** 1 Department of Neuroradiology, Friedrich-Alexander-Universität Erlangen-Nürnberg (FAU), Schwabachanlage 6, 91054 Erlangen, Germany; michael.knott@uk-erlangen.de (M.F.X.K.); philip.hoelter@uk-erlangen.de (P.H.); s.lang@uk-erlangen.de (S.L.); arnd.doerfler@uk-erlangen.de (A.D.); manuel.schmidt@uk-erlangen.de (M.A.S.); 2 Department of Psychiatry and Psychotherapy, Friedrich-Alexander-Universität Erlangen-Nürnberg (FAU), Schwabachanlage 6, 91054 Erlangen, Germany; timo.oberstein@uk-erlangen.de (T.O.); johannes.kornhuber@uk-erlangen.de (J.K.)

**Keywords:** dementia, Alzheimer’s disease, frontotemporal dementia, mild cognitive impairment, brain volumetry, pulsed arterial spin labeling

## Abstract

*Background*: Previous studies suggest that brain atrophy can not only be defined by its morphological extent, but also by the cerebral blood flow (CBF) within a certain area of the brain, including white and gray matter. The aim of this study is to investigate known atrophy patterns in different forms of dementia and to compare segmented brain volumetrics and pulsed arterial spin labeling (pASL) data to explore the correlation between brain maps with atrophy and this non-contrast-enhanced brain-perfusion method. *Methods*: Our study comprised 17 patients with diagnosed cognitive impairment (five Alzheimer’s disease = AD, five frontotemporal dementia = FTD, seven mild cognitive impairment = MCI) and 19 healthy control subjects (CO). All patients and controls underwent 4D-pASL brain-perfusion MR imaging and T1w MPRAGE. The data were assessed regarding relative brain volume on the basis of 286 brain regions, and absolute and relative cerebral blood flow (CBF/rCBF) were derived from pASL data in the corresponding brain regions. Mini-Mental State Examination (MMSE) was performed to assess cognitive functions. *Results*: FTD patients demonstrated significant brain atrophy in 43 brain regions compared to CO. Patients with MCI showed significant brain atrophy in 18 brain regions compared to CO, whereas AD patients only showed six brain regions with significant brain atrophy compared to CO. There was good correlation of brain atrophy and pASL perfusion data in five brain regions of patients with diagnosed FTD, especially in the superior temporal gyrus (r = 0.900, *p* = 0.037), the inferior frontal white matter (pars orbitalis; r = 0.968, *p* = 0.007) and the thalami (r = 0.810, *p* = 0.015). Patients with MCI demonstrated a correlation in one brain region (left inferior fronto-occipital fasciculus; r = 0.786, *p* = 0.036), whereas patients with diagnosed AD revealed no correlation. *Conclusions*: pASL can detect affected brain regions in cognitive impairment and corresponds with brain atrophy, especially for patients suffering from FTD and MCI. However, there was no correlation of perfusion alterations and brain atrophy in AD. pASL perfusion might thus represent a promising tool for noninvasive brain-perfusion evaluation in specific dementia subtypes as a complimentary imaging-based bio marker in addition to brain volumetry.

## 1. Introduction

Dementia is an overall definition of conditions and brain diseases causing a long-term and often steady decline in cognitive abilities and memory, which are severe enough to impair daily life functioning, often including emotional, social and behavioral deficits. The understanding of the biological mechanisms for developing dementia in the human nervous system is as yet widely incomplete.

There are a variety of biomarkers used for attempting to diagnose different forms of dementia and more sensitive monitoring of progression, even at an early stage of onset. In particular, Alzheimer’s disease (AD)—the most common cause of dementia (~70%)—and frontotemporal dementia (FTD) are well-examined conditions. Examples for biomarkers are cerebrospinal fluid biomarkers such as total tau, phosphorylated tau, and β-amyloid1–42 [1], or genetical biomarkers such as the APOE-ε4 allele as one of the strongest non-Mendelian genetic risk factors [2] for AD. Similarly, various gene mutations for the FTD spectrum are known. Correlatively, structural magnetic resonance imaging (MRI) can reveal circumscribed atrophy patterns of brain lobes. Reduced cerebral blood flow (CBF) seems to be associated with neurodegenerative progression due to reduced brain metabolism; however, it is not yet clear which one precedes the other. Therefore, CBF could be a surrogate marker of brain function (neurovascular coupling) [3].

Accordingly, arterial spin labeling (ASL), a relatively new method for analyzing brain perfusion and regional CBF, alongside dynamic susceptibility contrast perfusion or single photon emission computed tomography, has been suggested as an uprising biomarker to possibly detect early onset of dementia [4], which warrants regular consideration in the clinical setting. As opposed to other perfusion techniques, ASL is a completely noninvasive procedure without the demand for contrast agent or costly radioactive tracers. ASL scans are comparably short (approx. 4 min) and can be accomplished during the same examination of structural MRI, providing for an even more accurate diagnosis of the structure–function relationship [4]. Furthermore, MRI is easy to access and less expensive than other procedures, e.g., fluorodeoxyglucose (18F)– positron emission tomography (FDG-PET).

In this study, we compare morphological and functional MR neuroimaging of patients suffering from clinically diagnosed FTD, AD and MCI to determine regions of significant brain atrophy and correlating CBF and/or relative CBF (rCBF) via pulsed ASL (pASL). Unlike prior studies, data were divided into much smaller sections of brain regions by a comprehensive set of pre-defined brain structures including gray and white matter (286 regions) via an automated online application tool to give more detailed insight.

## 2. Materials and Methods

### 2.1. Participants

We examined seventeen subjects with different forms of cognitive impairment and nineteen control subjects, which altogether were recruited from the University Hospital Erlangen, Bavaria, Germany (i.e., psychiatric and neuroradiologic department at the University Hospital Erlangen, Bavaria, Germany). All participants provided written informed consent according to procedures approved by institutional review boards and the ethic-commission of Friedrich-Alexander-University Erlangen-Nürnberg (FAU) (231_12 Az; EudraCT: 2012-001583-29). The examination was performed at the Neuroradiology, University Hospital Erlangen. Five patients were clinically diagnosed with AD, five patients with FTD and seven patients with MCI. (For full demographic features, see Table 1).

### 2.2. Procedures

Clinical examination as well as structural and pASL-MRI scans were performed. The subjects underwent medical evaluation by psychiatrists comprising medical history, physical and neurologic examination, and neuropsychological testing. General cognitive ability was assessed by Mini-Mental State Examination (MMSE).

### 2.3. Structural MRI Acquisition

Imaging was performed on a 3 Tesla MR system (Siemens Trio Tim System, Siemens Healthineers, Erlangen, Germany), in a standardized supine position using a 12-Channel Head Coil (Siemens Healthineers, Erlangen, Germany). Structural MRI included following sequences: 3D FLASH MRI along 3 orthogonal directions to obtain scout views of the brain for initial positioning of MRI slices (voxel size: 1.6 × 1.6 × 1.6 mm, matrix size = 160 × 160, total acquisition time: 17 s); sagittal volumetric T1-weighted gradient echo MRI (MPRAGE) of the entire brain, TR/TE/TI = 1900/2.52/900 ms, 9° flip angle sequence with a spatially isotropic resolution of 1.0 mm^3^ (matrix size = 256 × 256, total acquisition time: 4:26 min); sagittal single slab 3D turbo spin echo sequence with slab selective, variable excitation pulse to obtain proton density and T2-weighted MRIs, TR/TE/TI = 5000/388/1800 ms, 176 contiguous slices (1 mm) covering the entire brain with a spatially isotropic resolution of 1.0 mm^3^ (matrix size = 256 × 259, total acquisition time 6:27 min); axial diffusion weighted imaging (DWI) with TR/TE = 3800/91 ms, a slice thickness of 5 mm, FOV = 230 mm (voxel size: 1.2 × 1.2 × 5.0 mm, matrix size = 192 × 192, total acquisition time 1:21 min); axial 3D fast low-angle gradient-echo sequence (SWIMR), TR/TE = 28/20 ms, FOV = 230 mm, slice thickness of 2 mm with a spatially anisotropic resolution of 0.7 × 0.6 × 2.0 mm (matrix size = 384 × 353, total acquisition time 5:26 min). To rule out intra- and extracranial stenosis, which could compromise arterial spin labeling technique we performed intra- and extracranial angiography and contrast-enhanced brain perfusion. Angiography MRI included: 3D time-of-flight MRA spoiled gradient echo (TOF) of the circulus arteriosus cerebri with TR/TE = 22/3.98 ms, 15° flip angle sequence with FOV = 220 mm, slice thickness/slice oversampling = 0.7 mm/25%, and bandwidth = 181 Hz/Px (voxel size: 0.7 × 0.4 × 0.7 mm, matrix size = 512 × 307, total acquisition time 5:37 min); contrast-enhanced MRA with a weight-adapted dose of Gadobutrol (Gadovist^®^, Bayer Healthcare, Leverkusen, Germany), 0.1 mL/kg) injected at a rate of 3 mL/s, TR/TE 43.46/1.56 ms, 25° flip angle, bandwidth 400 Hz/Px, 90 measurements (voxel size: 0.7 × 0.4 × 0.7 mm, matrix size = 256 × 179, total acquisition time 1:30 min); dynamic susceptibility contrast (DSC) MR perfusion with a second weight-adapted dose of Gadobutrol (0.1 mL/kg) injected at a rate of 5 mL/s, a T2* echo planar imaging (EPI) gradient recalled-echo sequence was used, TR/TE = 1840/32 ms, 90° flip angle, bandwidth 1346 Hz/Px, slice thickness = 6 mm, 19 sections (matrix size = 128 × 128, total acquisition time 1:39 min); postcontrast structural MRI included the following sequences: postcontrast axial T1 fast low-angle shot (FLASH) sequence with TR/TE = 309/4.92 ms, 90° flip angle (voxel size 0.5 × 0.5 × 5.0 mm, matrix size = 448 × 448, total acquisition time 2:41 min); postcontrast axial T1 volumetric interpolated brain examination (VIBE) 7 sequence with TR/TE = 3.93/1.5 ms, 9° flip angle (voxel size = 0.9 × 0.9 × 2.0 mm, matrix size = 320 × 320, total acquisition time 2:44 min).

### 2.4. pASL-Perfusion Image Acquisition

ASL was performed using a quantitative pulsed ASL technique called Proximal Inversion with Control of Off-Resonance Effects, or PICORE, before the administration of the contrast agent. We acquired asymmetric multi-slice pASL-MRI sequences with a slice-selective 90° pulse and spoiler gradient to initially saturate magnetization in the imaged slice. A wide inversion slab was then applied proximally using a 180° adiabatic RF pulse that inverts inflowing spins. The control sequence repeated the 90° spoiler saturation of static tissue in the imaged slice with nonselective off-resonance inversion pulse that is applied at the same frequency offset relative to the imaging slice as the tag, but in the absence of a slab-selective gradient (actual imaging parameters were as follows: TR 2500 ms, TE 11 ms, TI1 = 700 ms, saturation stop time = 1600 ms, TI2 = 1800 ms, flow limit = 100.0 cm/sec, field of view 192 mm, covering 14 slices with slice thickness 6 mm, gap = 1.5 mm, distance factor 25%, 101 measurements, matrix size = 64 × 64 and total acquisition time 4:22 min).

### 2.5. Brain Volumetry Analysis

**Figure 1:**
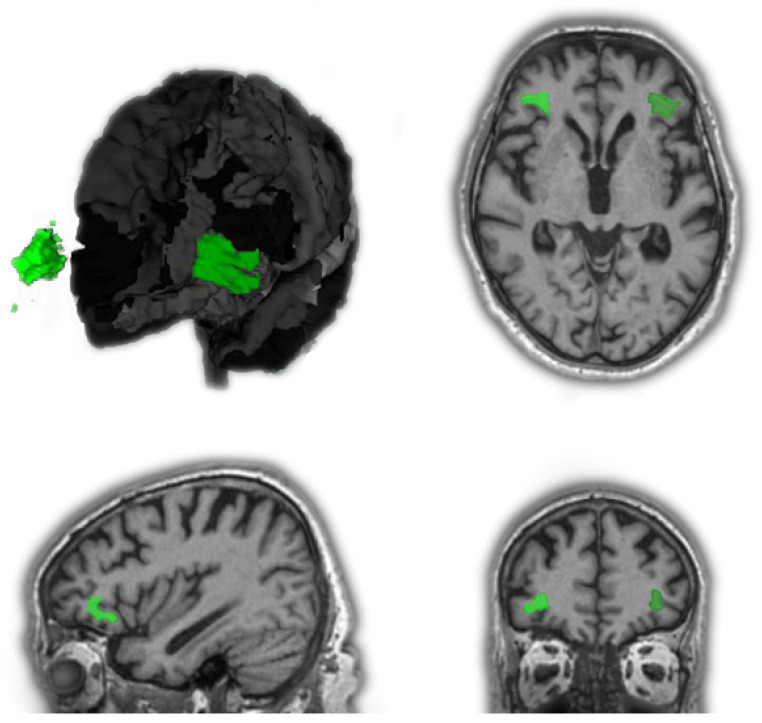
Views of the volumes of interest, exemplarily “inferior frontal white matter of the pars orbitalis (IFWMO)” (right: light green; left: dark green). Three-dimensional reconstruction with removed right brain hemisphere (except for the volume of interest) and partially removed frontotemporal brain segments of the left brain hemisphere, respectively, for better display. Images from top left, clockwise: 3D reconstruction, axial view, sagittal view and coronal view.

Volumetric data of segmented brain regions were obtained by using an online application called Brain Geodesic Positioning System—BrainGPS (AnatomyWorks, LLC, Baltimore, MD, USA)—to process brain MRI images and define various brain regions automatically [5]. It is based on an advanced multiple-atlas algorithm. Acquired T1w-MPRAGE-DICOM data were converted to 4D analyze format (with .hdr/.img file extension) by Dcm2analyze to eliminate personal information. They were then uploaded to the application and submitted to obtain a 286-label segmentation of the T1w-MPRAGE scan for ROI information (T1-MultiAtlas) using the XSEDE Computational Anatomy Science Gateway. The results included segmentation of 286 regions as well as summary statistics of the structures. Three different types of volume reports are provided in this analysis at different granularity levels: raw volumes (mm^3^) in the native patient coordinates, normalized brain size (tissue, ventricles and sulci as the denominator), and linear normalization to the MNI space [6]. For volumetry statistics, the normalized brain size at granularity Level 5 (Tree Type I) [7] was used. T1 MultiAtlas results were additionally managed, inspected, and stored in a novel 3D database system called BrainKnowledge (AnatomyWorks, LLC, Baltimore, MD, USA) to visualize all examined regions of interest in the MNI space (see Figure 1).

### 2.6. pASL Perfusion Data Analysis

Acquired pASL-DICOM data were also converted to 4D analyze. The converted data were uploaded simultaneously with the T1 MultiAtlas of each subject and submitted for processing and ROI analysis. Several default parameters for CBF quantification, such as blood T1, brain/blood partition coefficient, and labeling efficiency were assumed by this processing and not modified [8]. Output data included absolute CBF map in native ASL space, rCBF map in native ASL space with CBF values relative to the whole brain CBF average, a text file that contains motion vectors from realignment, a text file that contains the information of the dataset for reference, CBF and rCBF maps in MPRAGE space, CBF and rCBF maps in standard MNI space, segmented brain image in MPRAGE space, a text file that contains CBF values in the ROIs, and a brain mask that shows hypo- and hyperperfusion regions.

### 2.7. Statistical Analysis

Statistical analysis was performed using IBM SPSS Statistics (version 19.0.0.1; IBM SPSS-Software, Ehningen, Germany). An independent t-test was used to calculate significant brain atrophy in any of the processed 286 brain regions by comparing quantitative values of the brain volumes of subjects with the same diagnosed cognitive impairment against the control group. Significance level was defined as *p* < 0.05. Normal distribution was tested using the Kolmogorov–Smirnov test. The assumption of homogeneity of variance was tested using Levene’s test of equality of variances. We informally accounted for multiple comparisons as previously suggested [9]. For brain regions demonstrating significant atrophy, the bivariate correlations procedure was used to compute Pearson’s correlation coefficient, Spearman’s rho, and Kendall’s tau-b with their significance levels for brain volumetry (mm^3^), CBF (mL/100 g/min), and rCBF (mL/100 g/min) to identify linear correlation. The significance level was defined as *p* < 0.05 (two sided).

## 3. Results

### 3.1. Results of Brain Regions with Significant Atrophy

**Figure 2:**
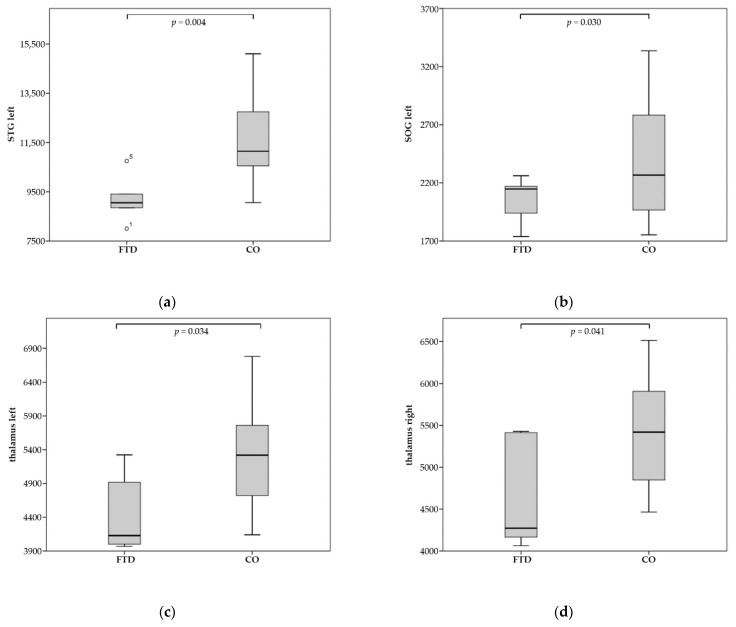

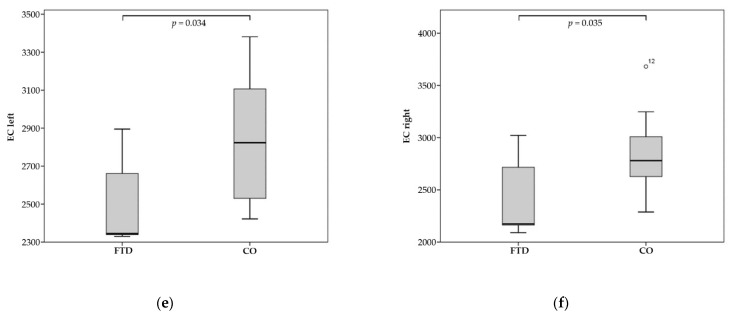
Significant atrophy of regions for FTD compared to CO (control)—volumes in mm^3^: (**a**) left STG; (**b**) left SOG; (**c**) left thalamus; (**d**) right thalamus; (**e**) left EC; (**f**) right EC.

Patients diagnosed with FTD demonstrated significant brain atrophy in 43 brain regions (of a total of 286) compared to the CO group. As anticipated, there was significant atrophy for frontotemporal brain regions such as the right middle fronto-orbital gyrus (MFOG), the left and right inferior frontal gyrus pars orbitalis (IFGO), the left and right middle frontal gyrus (dorsal prefrontal cortex) (MFG-DPFC), the left and right gyrus rectus (RG), the left and right inferior frontal white matter of the pars orbitalis (IFWMO), the left and right superior temporal gyrus (STG), the left middle temporal gyrus (MTG), the left and right amygdala, and the left hippocampus. Additionally, there was a significantly reduced volume in the left and right external capsule (EC) and the left and right thalamus (for full results, see Table 2 and Figure 2).

**Figure 3:**
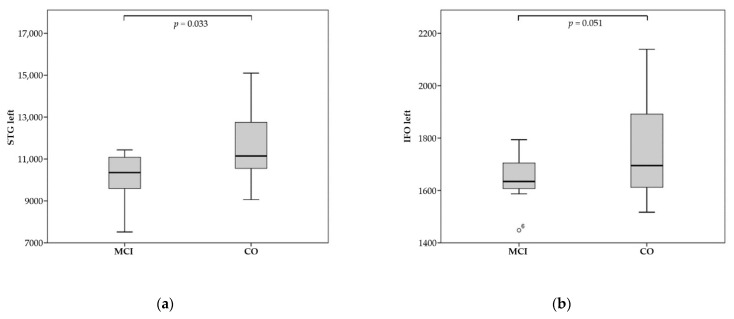
Significant atrophy of regions for MCI compared to CO (control)—volumes in mm^3^: (**a**) left STG; (**b**) left IFO.

MCI patients showed significant brain atrophy in 18 brain regions. There was particularly high significance for the left and right STG, the right lateral fronto-orbital white matter (LFOWM), and the left rostral anterior cingulate white matter (rostralWMACC) (for full results, see Table 3 and Figure 3).

Patients diagnosed with AD presented six brain regions with significant atrophy. Understandably, there was especially high significance for hippocampal brain regions, such as the right hippocampus and the right hippocampal cingulum (CGH). A reduced volume of the right middle temporal white matter (MTWM) was measured (for full results, see Table 4).

### 3.2. Results of Statistical Correlation between Brain-Volume and pASL

There was good correlation of brain atrophy with reduced pASL perfusion data in five brain regions of patients with diagnosed FTD. Patients indicating signs of MCI demonstrated correlation in one brain region, while patients with diagnosed AD revealed no good correlation.

#### 3.2.1. Patients with FTD

**Figure 4:**
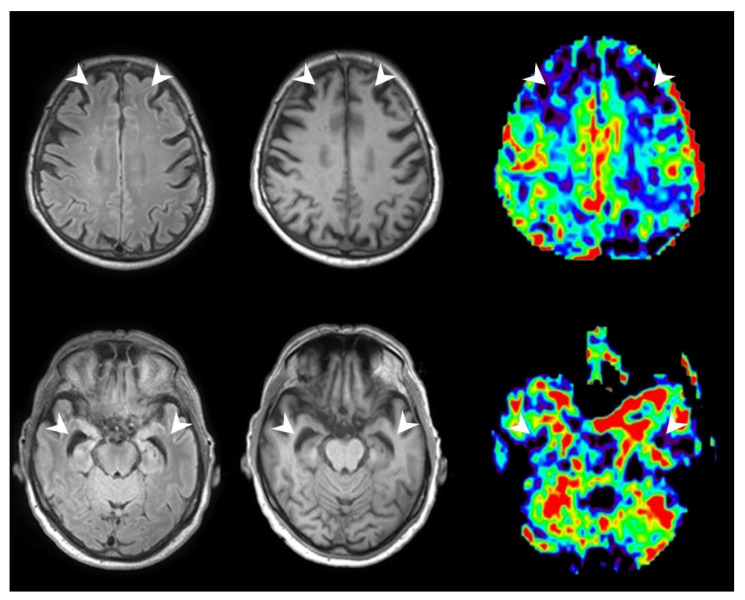
A patient with diagnosed FTD and corresponding pASL perfusion maps showing reduced CBF in the frontotemporal regions. From left to right: FLAIR Sequence, T1w Sequence, pASL Sequence; perfusion data were visualized with OLEA SPHERE^®^ 2.3 (Olea medical, La Ciotat, France)(white arrows indicating regions of atrophy and hypoperfusion).

Of the 43 brain regions, five displayed good linear correlation of significant brain atrophy and a reduced CBF—in two cases, additionally rCBF—of the pASL data. The significant results for FTD patients can be summarized as follows:

We observed a good positive correlation between brain volume and CBF/rCBF for the left STG (Spearman’s rho = 0.900, *p* = 0.037). The right IFWMO demonstrated very good correlation between CBF and volume (Pearson = 0.968, *p* = 0.007; Spearman’s rho = 0.900, *p* = 0.037). After having excluded one patient with negative pASL values, the merged observation of both thalami showed good positive correlation of CBF/rCBF data with brain volume (CBF: Pearson = 0.810, *p* = 0.015; Spearman’s rho = 0.810, *p* = 0.015; Kendall Tau-b = 0.643, *p* = 0.026; rCBF: Pearson = 0.722, *p* = 0.043). Ultimately, there was good positive linear correlation between volume and CBF for the right EC (Spearman’s rho = 0.900, *p* = 0.037). For exemplary imagery, see Figure 4.

#### 3.2.2. Patients with MCI

Of the 18 brain regions, one displayed a good positive linear correlation between significant brain atrophy and a reduced rCBF of the pASL data. The left inferior fronto-occipital fasciculus (IFO) demonstrated a good correlation between rCBF and brain volume (Spearman-Rho = 0.786, *p* = 0.036).

#### 3.2.3. Patients with AD

Of the six volume-reduced brain segments, none demonstrated a good positive linear correlation of significant brain atrophy and reduced CBF or rCBF of the pASL data.

## 4. Discussion

Prior studies suggest that certain atrophy patterns accompany hypoperfusion for different kinds of dementia, e.g., the prefrontal cortex, precuneus and hippocampal lobes in AD [10], and the thalami and reasonably the frontotemporal lobes in FTD [11]. Consequently, further reviews indicate, for example, hippocampal atrophy as a risk factor for progression to dementia in patients suffering from mild cognitive impairment (MCI) [12]—a prodromal state of AD. Sets of atrophy patterns might help to differentiate between different kinds of dementias, which sometimes are not easily diagnosed due to overlapping symptoms. For example, AD accounts for ~30% of cases presenting with a clinical diagnosis of primary progressive aphasia (PPA) or behavioral-variant frontotemporal dementia (bvFTD) [13]. Accuracy of discrimination at an early or even asymptomatic state of dementia could support appropriate initial target-oriented medical care, e.g., medication with acetylcholinesterase inhibitors.

We found a good correlation of formerly described atrophy patterns for FTD, MCI, and AD compared to CO. Consistently with the pathophysiology of FTD, we found significant brain atrophy in the frontotemporal brain structures of patients with FTD (MFG-DPFC, IFGO, MFOG, RG, and STG) since frontotemporal lobar degeneration involves the deterioration of neurons in the gray matter of the frontotemporal lobes [14]. Volume reduction in IFG and STG—being classic language areas (Broca’s and Wernicke’s area)—is of great significance for FTD, especially in non-fluent-variant primary progressive aphasia (nfvPPA) and semantic-variant primary progressive aphasia (svPPA). Atrophy in the mesial temporal lobe is commonly seen in FTD patients [15]; in more detail, we could confirm amygdalar atrophy, which has been neuropathologically identified before [16]. The amygdala executes primary roles in the accumulation and formation of memories related to emotional events [17]; therefore, volume reduction of the amygdala might interfere with memory recall that is strengthened by emotion. Hippocampal atrophy is not solely specific for FTD and is rather pathognomonic for AD [18], which makes hippocampal atrophy per se an uncertain imaging biomarker for FTD. As stated in prior studies, we can verify significant atrophy of the thalamus, which is a well-examined characteristic for the whole FTD spectrum [11,19]. Thalamic as well as amygdalar atrophy in turn suggest a loss of cortical projection fibers, which might explain the reduced volume of the caudate tail, putamen, and globus pallidus—all of them connected to the thalamus via axonal pathways.

Striatal degeneration in patients with FTD has been well demonstrated [20], explicitly with the putamen being associated with reinforcement learning and motor control, including articulation and speech [21]. Complementing these results, we found significant volume loss of the substantia nigra as an integral component of the basal ganglia circuitry for motor and limbic functions. Degeneration of the basal ganglia is a well-known aspect of neurodegenerative diseases, which might explain the progressive development of motor deficits in FTD patients, including parkinsonism and motor neuron diseases [15]. A number of studies found subcortical involvement in FTD patients confirming these findings [22,23]. Hence, it is not surprising that we also found significant white matter-atrophy in the corona radiatia, cerebral peduncles, ECs and frontal brain regions (IFWMO, IFO), which contain descending (motor) nerve tracts and association fibers among others, alluding to an axonal degeneration as a consequence or as a precursor of (sub)cortical atrophy.

MCI represents a preclinical transitional stage between healthy aging and dementia. Hypoperfusion and hypometabolism in the temporoparietal cortex and mesial temporal lobe atrophy, particularly in the rhinal cortex, have been described [24,25]. This explains our findings of atrophy in the right hippocampus and parahippocampal gyrus. Additionally, we found significant atrophy in the MTG and STG. Maybe due to the connection of the anterior cingulate with the hippocampus, there is significant adjacent white matter atrophy (subcallosalWM-ACC, rostralWM-ACC) [26]. Since there is no verified atrophy in other associated cortical structures to these white matter fibers, this could support the theory that hippocampal atrophy is a precursor leading to white matter degeneration of the corresponding axons. There are studies stating that anterior cingulate cortex disruptions can be found in AD and MCI patients [27,28]. High-level functions for the ACC such as focused problem solving and error recognition have been described [26], which might be unrecognized in the diagnosis of MCI. Our finding of atrophy in the left IFO seems complex. The inferior fronto-occipital fasciculus contains fibers that connect the frontal lobe with the posterior part of the parietal and temporal lobes. In the temporal lobe, the inferior occipitofrontal fasciculus is associated with the inferior longitudinal fasciculus, which interconnects the occipital cortex (lingula, cuneus, and lateral surface) with the cortex of the superior, middle and inferior temporal (and possibly the hippocampal) gyri. This complex pathway of connections may account for a degeneration of associated fibers to the hippocampus and temporal gyri in the IFO. Defects in the IFO can cause visual hallucinations and global aphasia, and there is also evidence that trauma to the IFO can cause amnesia and cognitive impairment [29,30]. Additionally, we found atrophy of the left mammillary body, which in turn can be present in AD [31]—MCI being a prodromal state of AD. Previous studies have found the mammillary bodies to be structurally connected to memory function, partially linked to the hippocampus via pathways, which explains cognitive deficits in patients with Wernicke–Korsakoff syndrome [32].

Numerous studies have convincingly documented hippocampal atrophy as well as atrophy of temporal brain regions (STG, MTWM) for patients diagnosed with AD in comparison to aging controls [33], which we can reproduce. Volume reduction in the cingulum (CGH) could be a consequence or precursor, as it is connected to the hippocampal formation (e.g., subiculum) via projection fibers [34]. Our findings of adjacent white matter atrophy in our MCI cohort insinuate that atrophy in the cingulum could be consequential; however, we cannot reproduce significant atrophy in subcallosal WM-ACC or rostral WM-ACC for AD patients, which makes this a rather questionable conclusion. As expected, and in contrast to FTD patients, we could not find significant proof of striatal atrophy [35], except for the left caudate tail. The caudate tail is not well examined, although a prior study has suggested that there is especially relevant atrophy in the left caudate head and body for AD patients compared to CO, yet seems to be more present in the FTD spectrum [36]. This in turn suggests striatal degeneration to be a good diagnostic biomarker for FTD [36,37]. Atrophy of SOG is rarely described and might be present in patients suffering from atypical AD. As these patients do not primarily present with memory impairment but atypical symptoms, e.g., in the fields of language or vision [38], posterior cortical atrophy seems to play an important role. However, we did not observe significant atrophy of parietal lobe structures such as the precuneus, which has been described for (a)typical and early-onset AD patients [39]. Contrary to former studies, we found that the right hemisphere seems to be more affected by the disease than the left one in our AD cohort, although left–right asymmetry, especially of the hippocampus, receives less research attention [40].

A strength of this study is the fine segmentation for pASL perfusion data into 286 brain regions. FTD naturally shows frontotemporal patterns of hypoperfusion, which we could reproduce by a good positive correlation of volume and cerebral blood flow (STG, IFWMO) [41]. To the best of our knowledge, there are no other studies confirming reduced perfusion rates in atrophied thalami and the right EC in FTD patients so far. Thalamic atrophy, as discussed above, is a well-examined subject matter for FTD patients, so it is not surprising that we found reduced pASL perfusion rates. Loss of white matter microstructural integrity of the EC in FTD has been measured via DTI previously [42]. The clinical benefit of this particular finding, however, remains doubtful. On the one hand, our findings of a rather low correlation between volume and perfusion overall supports studies which have doubted the beneficial use of ASL in comparison to FDG-PET in the diagnosis of FTD [43,44]. On the other hand, there are studies confirming a good correlation of ASL perfusion with FDG-PET [41,45]. These conflicting findings could be best explained by the heterogeneity of relatively small sample populations and differing ASL techniques [46]. Our finding of a good correlation for volume loss in the IFO in MCI patients has not yet been discussed. Again, the clinical importance of this finding remains unclear, although degeneration or defects in the IFO (as described above) could produce severe symptoms. The fact that we did not find good correlation in the AD spectrum could be due to the lower sample size in that subgroup, as well as their mild clinical symptoms. Lastly, there is discussion around if atrophied brain regions may demonstrate paradox hypermetabolism and hyperperfusion as a last “distress call”, especially in patients with MCI and AD [47].

As stated, our present study may be limited due to its small sample of participants, which might explain why we only found relatively few correlations of reduced CBF/rCBF with volume loss. Furthermore, there was no differentiation between subtypes of the FTD, MCI, and AD spectrum. Most of our AD patients were mildly affected (MMSE > 21) [48,49,50], which could explain the lack of a good correlation of hypoperfusion with atrophied brain regions, as well as the lack of atrophy in the parietal lobes. However, our results are in line with the formerly described atrophy patterns of other brain regions, the hippocampus being one of the earliest sites of pathological involvement, even in mild AD [34]. Image acquisition of pASL data in the elderly is sometimes difficult, as lacking cooperation and compliance during an MRI examination of cognitively impaired patients might lead to reduced image quality by motion artifacts. Yet, motion artifacts can be ruled out for structural MRI.

There are hints that cerebral hypoperfusion precedes neurodegeneration in large population-based studies [51]. It has been suggested that lower perfusion is often attributed to neurodegeneration and can indicate neuronal dysfunction and synaptic failure. The first signs of neurodegeneration are likely to occur years before the diagnosis of dementia, and cerebral perfusion may consequently fall well before clinical symptoms of dementia arise. Unfortunately, we cannot deliver any answers to the question of whether reduced blood flow in certain brain regions leads to a decline in brain volume, or vice versa, volume loss and reduced metabolism leading to a downgrading of the need for blood supply. This would need longitudinal studies and was not the main subject matter of this study.

The aim of our study was to compare brain perfusion patterns regarding hypoperfusion with sophisticated brain volumetric data regarding atrophy. We found significant correlations of brain areas affected by hypoperfusion and atrophy. Thus, our results support the idea that brain perfusion evaluation by pASL and volumetric assessment of brain parenchyma can deliver complimentary MR imaging-based biomarkers in early detection and follow-up of dementia. To the best of our knowledge there are no studies confirming ASL hypoperfusion in atrophied thalami of the FTD spectrum so far, which should be subject to further investigation. Clinically, pASL can easily be established in routine MRI procedures and would not involve intravenous administration of contrast agent or radiopharmaceuticals, which not only preserves multimorbid patients (e.g., severe kidney failure) but also comes in at a lower cost than, for example, SPECT brain perfusion, which is often used instead.

It has been suggested that atrophic patterns are not necessarily coherent with hypoperfusion patterns, hinting at varying pathological mechanisms in the development of dementia disorders [52]. This in turn gives rise to the question of if combined patterns of pASL and volumetry could be a more precise MR imaging-based biomarker to differentiate between different forms of dementia. For this purpose, further studies are needed. Moreover, prospective ultra-high-field (e.g., 7 Tesla MRI) imaging studies may give more detailed insight into atrophy and perfusion changes of the brain in elderly and cognitively impaired patients.

## 5. Conclusions

pASL can detect affected brain regions in cognitive impairment and corresponds with brain atrophy, especially for patients suffering from FTD and MCI. However, there was no correlation of perfusion alterations and brain atrophy in AD. This supports the theory that pASL could provide complimentary imaging biomarkers for identifying affected brain regions in addition to brain volumetry in specific dementia subtypes—particularly in patients with FTD and MCI—maybe even at an early stage of disease.

## Figures and Tables

**Table 1:** Demographic features of patients and controls.

	CO	AD	FTD	MCI
No.	19	5	5	7
Sex (M:F)	7:12	4:1	1:4	4:3
Age (years)	57.1 (46–78)	63.6 (50–77)	69.8 (62–79)	63 (54–78)
MMSE	30	22.4 (10–27)	24.6 (14–30)	27.4 (25–29)

Note: AD indicates Alzheimer’s disease; FTD, frontotemporal dementia; MCI, mild cognitive impairment; CO, control subjects; MMSE, Mini-Mental State Examination.

**Table 2:** Significant atrophy in brain regions of interest—FTD vs. CO.

Brain Region of Interest	Mean Volume in FTD (in mm^3^)	Mean Volume in CO (in mm^3^)	Level of Significance (*p*)
MFG-DPFC left	9525.6	11,480.0	0.053
MFG-DPFC-right	9763.6	11,947.1	0.031
IFGO left	2755.0	3347.1	0.023
IFGO right	2062.2	2622.6	0.002
MFOG right	3534.4	4028.0	0.041
RG left	3770.2	4625.9	0.026
RG right	3747.6	4691.5	0.006
STG left	9212.4	11,653.5	0.004
STG right	8948.2	10,913.4	0.004
STG-pole left	4168.4	5227.8	0.013
AG left	5866.8	6770.3	0.048
SOG left	2051.2	2395.6	0.030
Insula left	5104.8	6127.6	0.038
Insula right	5202.0	6141.0	0.024
Amygdala left	1071,8	1497,9	0.000
Amygdala right	1217.2	1630.7	0.006
Hippocampus left	2868.4	3442.0	0.022
Putamen left	2886.0	3684.6	0.001
Putamen right	3152.0	3876.3	0.002
GP left	1124.2	1340.4	0.025
Thalamus left	4468.4	5237.5	0.034
Thalamus right	4668.8	5348.7	0.041
NucAccumbens right	604.6	730.8	0.049
Snigra left	261.0	318.8	0.026
Snigra right	210.8	256.3	0.048
CP left	1536.2	1858.2	0.026
CP right	1623.4	1916.5	0.050
ICP left	631.4	761.0	0.036
SCR left	13,355.8	15,739.9	0.044
PCR left	5040.6	6124.4	0.013
PCR right	4243.2	5156.5	0.036
EC left	2514.0	2862.1	0.034
EC right	2433.0	2821.2	0.035
IFO left	1534.8	1765.8	0.024
IFO right	1561.2	1787.8	0.024
SS right	3210.8	4012.9	0.009
CI right	264.2	374.7	0.003
Basal Forebrain left	462.6	536.5	0.044
IFWMO left	1818.8	2283.6	0.008
IFWMO right	1543.2	1881.6	0.010
Caudate-tail left	273.4	338.1	0.030
Caudate-tail right	253.8	347.0	0.022
Fimbria right	16.4	36.5	0.011

Note: FTD indicates frontotemporal dementia; CO, control subjects.

**Table 3:** Significant atrophy in brain regions of interest—MCI vs. CO.

Brain Region of Interest	Mean Volume in MCI (in mm^3^)	Mean Volume in CO (in mm^3^)	Level of Significance (*p*)
SFG-pole left	2000.3	2290.2	0.039
STG left	10,094.4	11,653.5	0.033
STG right	9364.7	10,913.4	0.009
MTG right	12,041.7	13,891.3	0.030
MTG-pole left	2108.6	2460.1	0.048
PHG right	1059.1	1208.4	0.038
Hippocampus right	3187.7	3510.2	0.016
Basal Forebrain left	240.9	277.9	0.059
Basal Forebrain right	225.1	277.2	0.006
SCP left	736.0	831.8	0.011
Pons left	247.1	284.9	0.029
IFO left	1642.7	1765.8	0.051
Mammillary left	80.9	100.0	0.040
LFOWM right	1523.4	1683.6	0.002
RGWM left	1556.4	1787.8	0.052
STWM right	7752.7	8730.2	0.050
rostralWM-ACC left	65.1	141.2	0.002
subcallosalWM-ACC right	60.9	88.9	0.044

Note: MCI indicates mild cognitive impairment; CO, control subjects.

**Table 4:** Significant atrophy in brain regions of interest—AD vs. CO.

Brain Region of Interest	Mean Volume in AD (in mm^3^)	Mean Volume in CO (in mm^3^)	Level of Significance (*p*)
STG right	9516.6	10,913.4	0.056
SOG right	1916.4	2465.6	0.008
Hippocampus right	2873.0	3510.2	0.025
CGH right	1182.6	1456.6	0.036
MTWM right	7245.6	8682.2	0.045
Caudate-tail left	259.4	338.1	0.013

Note: AD indicates Alzheimer’s disease; CO, control subjects.

## Data Availability

Not applicable.

## Abbreviations

ACR	anterior corona radiata
AD	Alzheimer’s disease
AG	angular gyrus
ASL	arterial spin labeling
bvFTD	behavioral-variant frontotemporal dementia
CBF	cerebral blood flow
CGH	hippocampal cingulum
CI	claustrum complex
CP	cerebral peduncle
EC	external capsule
FDG-PET	fluorodeoxyglucose (18F)– positron emission tomography
FTD	frontotemporal dementia
GP	globus pallidus
ICP	inferior cerebellar peduncle
IFGO	pars orbitalis of the inferior frontal gyrus
IFO	inferior fronto-occipital fasciculus
IFWMO	pars orbitalis of the inferior frontal white matter
LFOWM	whiter matter of the lateral fronto-orbital gyrus
Mammillary	mammillary body
MCI	mild cognitive impairment
MFG-DPFC	middle frontal gyrus (dorsal prefrontal cortex)
MFOG	middle fronto-orbital gyrus
MMSE	Mini-Mental State Examination
MRI	magnetic resonance imaging
MTG	middle temporal gyrus
MTG-pole	pole of the middle temporal gyrus
MTWM	white matter of the middle temporal gyrus
nfvPPA	non-fluent variant primary progressive aphasia
NucAccumbens	Nucleus accumbens
pASL	pulsed arterial spin labeling
PCR	posterior corona radiata
PHG	parahippocampal gyrus
PPA	primary progressive aphasia
rCBF	relative cerebral blood flow
RG	gyrus rectus
RGWM	white matter of the gyrus rectus
rostralWM-ACC	white matter of the rostral anterior cingulate gyrus
SCP	superior cerebellar peduncle
SCRSFG-pole	superior corona radiatapole of the superior frontal gyrus
Snigra	substantia nigra
SOG	superior occipital gyrus
SS	sagittal stratum (includes inferior longitudinal fasciculus and inferior fronto-occipital fasciculus)
STG	superior temporal gyrus
STG-pole	pole of the superior temporal gyrus
STWM	white matter of the superior temporal gyrus
subcallosalWM-ACC	white matter of the subcallosal anterior cingulate gyrus
svPPA	semantic-variant primary progressive aphasia
